# Dynamic Changes in Anthocyanin Accumulation and Cellular Antioxidant Activities in Two Varieties of Grape Berries during Fruit Maturation under Different Climates

**DOI:** 10.3390/molecules27020384

**Published:** 2022-01-07

**Authors:** Liuwei Qin, Hui Xie, Nan Xiang, Min Wang, Shouan Han, Mingqi Pan, Xinbo Guo, Wen Zhang

**Affiliations:** 1 Engineering Research Center of Starch and Vegetable Protein Processing Ministry of Education, Guangdong Province Key Laboratory for Green Processing of Natural Products and Product Safety, School of Food Science and Engineering, South China University of Technology, Guangzhou 510640, China; qlw13710614008@163.com (L.Q.); nanxiang0908@163.com (N.X.); 2 Key Laboratory of Genome Research and Genetic Improvement of Xinjiang Characteristic Fruits and Vegetables, Research Institute of Horticulture, Xinjiang Academy of Agricultural Sciences, Urumqi 830091, China; xhxjnky@163.com (H.X.); wangmin_807032699@163.com (M.W.); hanshouan@163.com (S.H.); panmq3399@sohu.com (M.P.)

**Keywords:** different climates, grape berry development, anthocyanins, antioxidant capacity

## Abstract

As popularly consumed fruit berries, grapes are widely planted and processed into products, such as raisins and wine. In order to identify the influences of different climatic conditions on grape coloring and quality formation, we selected two common varieties of grape berries, ‘Red Globe’ and ‘Xin Yu’, for investigation. Grapes were separately grown in different climates, such as a temperate continental arid climate and a temperate continental desert climate, in Urumqi and Turpan, China, for five developmental stages. As measured, the average daily temperature and light intensity were lower in Urumqi. Urumqi grape berries had a lower brightness value (L*) and a higher red-green value (a*) when compared to Turpan’s. A RT-qPCR analysis revealed higher transcriptions of key genes related to anthocyanin biosynthesis in Urumqi grape berries, which was consistent with the more abundant phenolic substances, especially anthocyanins. The maximum antioxidant activity in vitro and cellular antioxidant activity of grape berries were also observed in Urumqi grape berries. These findings enclosed the influence of climate on anthocyanin accumulation and the antioxidant capacity of grapes, which might enlarge our knowledge on the quality formation of grape berries and might also be helpful for cultivating grapes with higher nutritional value.

## 1. Introduction

The grape (*Vitis vinifera* L.), with carbohydrates, organic acids, and minerals, is one of the most famous fruits around the world. Furthermore, grapes have also been shown in previous studies to contain valuable bioactive substances, including phenolic acids, flavonoids, and anthocyanins [1]. Anthocyanins are one of the main pigments that constitute the pericarp color in grapes [2]. Anthocyanins can protect plants from visible and ultraviolet damage and pests, thereby improving fruit quality [3,4,5]. Different epidemiological and preclinical studies have confirmed that food rich in phenolic substances, such as anthocyanins, can reduce the risk of chronic diseases in humans [6]. Therefore, bioactive substances and color are important indicators to measure the quality and maturity of grapes.

As reported, the concentration and composition of anthocyanins in grape berries are affected by environmental factors, including temperature, light, and moisture [7]. For example, the contents of anthocyanins in most grape varieties enhance with increasing light intensity, but overexposure leads to sunburn in berries and inhibits anthocyanin accumulation in some varieties [8]. Light promotes the compositions of both proanthocyanins and anthocyanins in grape pericarp with the relative upregulation of *LAR1*, *LAR2*, and *ANR* genes [9]. Similarly, the appropriate temperature can also promote the synthesis of anthocyanins, whereas the contents of anthocyanins are reduced under extreme high temperatures [10]. Low temperatures (10–11 °C) at night promoted anthocyanin synthesis in ‘Corvina’ grapes during veraison, and the transcriptional expressions of *CHS3*, *F3H1*, *MYBA1*, and *UFGT* were enhanced [11]. Temperature also affects anthocyanin content by regulating the metabolism of carbohydrates. The cooperation between low temperature and soluble compounds promoted the synthesis of anthocyanins in ‘Aki Queen’ grapes [12]. In addition, some studies have also confirmed that excessive high temperatures intensified the decomposition of anthocyanins in grape berries [13].

Urumqi and Turpan are the two principal grape-producing areas in Xinjiang, China, and ‘Red Globe’ (RG) and ‘Xin Yu’ (XY) grapes are the two regular cultivated varieties. Urumqi has a temperate continental arid climate, whereas Turpan has a temperate continental desert climate. Recently, researchers reviewed the influences of climate on grape maturity and quality, especially the effects from high temperatures [14]. How different climates affect the coloration of grapes during development has not been investigated yet. Furthermore, the influence of berry coloring on its quality is even less reported. In this study, we compared the color of grape berries and phenolics, including anthocyanin accumulation, as well as the cellular antioxidant activity of two grape varieties during five growth periods in Urumqi and Turpan, in order to figure out the influence climate has on grape coloring and quality formation. This study might enhance our knowledge on how climate influences the quality development of grapes.

## 2. Results and Discussion

### 2.1. Climatic Conditions in Urumqi and Turpan

Daily temperature, light intensity, and humidity in Urumqi and Turpan from 2 July 2018 to 14 September 2018 were recorded, as in Supplementary Figure S1. The average daily temperature in Turpan and Urumqi fluctuated around 40 °C and 30 °C, the average daily light intensity fluctuated around 800 μmol·m^−2^·s^−1^ and 500 μmol·m^−2^·s^−1^, and the average daily humidity fluctuated around 40% and 50%, respectively. As determined, Turpan had a higher temperature, a higher light intensity, and lower humidity when compared with Urumqi.

### 2.2. Color Difference Values of Grape Berries in Urumqi and Turpan

The color difference values of Red Globe (RG) and Xin Yu (XY) grape berries in Urumqi and Turpan during the five developmental stages (S1, S2, S3, S4, S5) are shown in Supplementary Table S1. The two different varieties of grape berries had more coloring with each development stage in the Urumqi and Turpan regions, but grape berries in Turpan had less coloring than those in Urumqi at each developmental stage (Supplementary Figure S2). With the elongation time of berry development, the L* values of berries were significantly reduced, and the Urumqi grape berries had lower L* values when compared with the Turpan-grown berries. L* values also varied in different species, as RG grape berries have a lower value than XY grape berries. The a* values of Urumqi berries increased in the initial stage and then decreased when the grapes matured, whereas the a* values of Turpan berries showed an upward tendency during development. Among RG grapes, the a* values of Urumqi berries were higher than Turpan berries at the three early developmental stages. For XY grapes, the a* values of Urumqi berries were higher than Turpan berries at S2, S3, and S4. Distinct changeable rules in the a* values between the two varieties were shown in different regions. The ΔE and b* values of berries gradually decreased, and the b* values of Urumqi berries were lower than Turpan berries during the late stage of grape development.

The color difference value is an important indicator for evaluating the color of grape berries. The smaller L* values and larger a* values illustrate the deeper degree of coloring of grape peels. In this study, the redness of berries deepened as grapes matured (Supplementary Figure S2). Urumqi grape berries were obviously redder than Turpan grape berries, and the a* values of Urumqi berries were larger. As analyzed, different climates not only influenced L* values but also changed the tendency of a* values during the development of grape berries. Previous studies have also shown that the lower the root temperature and phosphorus content, the deeper the redness and the lower the brightness of red lettuce leaves, the larger the a* values, and the smaller the L* values [15]. In our results, grape berries grown in the Urumqi region with lower temperatures correspondingly possessed lower L* values and higher a* values during development as compared to Turpan.

### 2.3. Gene Expression of Grape Berries in Urumqi and Turpan

**Figure 1:**
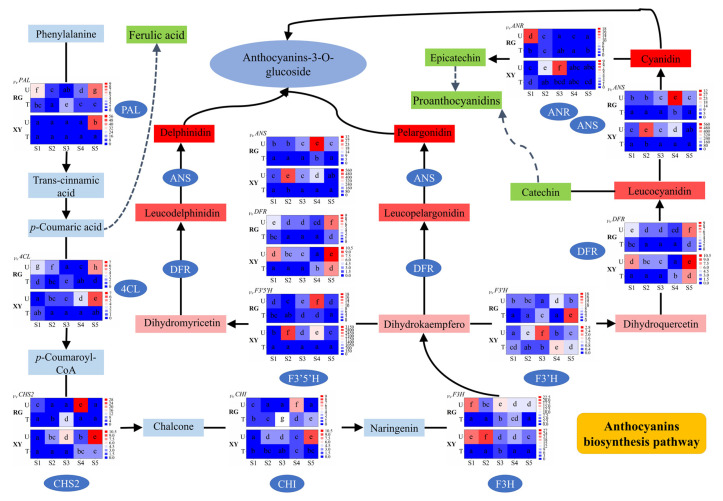
Gene relative expression levels of the anthocyanin biosynthesis pathway in grape berries during the five developmental stages in Urumqi and Turpan. The data presented are as mean ± SD of triplicates. *PAL* encodes phenylalanine ammonia lyase; *4CL* encodes 4-coumarate-CoA ligase; *CHS2* encodes chalcone synthase; *CHI* encodes chalcone isomerase; *F3H* encodes flavanone 3-hydroxylase; *F3′H* encodes flavonoid-3′-hydroxylase; *F3′5′H* encodes flavonoid-3′5′-hydroxylase; *DFR* encodes dihydroflavonol-4-reductas; *ANS* encodes anthocyanin synthase; and *ANR* encodes anthocyanidin reductase. RG stand for ‘Red Globe’ grapes; XY stand for ‘Xin Yu’ grapes; U stands for Urumqi; T stands for Turpan. Bars with different letters differ significantly at *p* < 0.05.

Gene expression studies on the main genes involved in the anthocyanin biosynthesis of grape berries from Turpan and Urumqi were conducted by RT-qPCR. The anthocyanin biosynthesis pathway and relative expression levels of *PAL*, *4CL*, *CHS2*, *CHI*, *F3H*, *F3′H*, *F3′5′H*, *DFR*, *ANS*, and *ANR* genes are shown in Figure 1. The average expression rates of RG and XY grape berries’ genes were calculated as the ratio of the relative expression of these key genes at each stage and Turpan S1. *PAL* is the first gene for the synthesis of anthocyanins in grape berries. In general, the expression of *PAL* in each sample unanimously downregulated at first but followed with an upregulating trend in later stages. Overall, the expression of *4CL* in Urumqi berries reached its maximum at S4, whereas it was downregulated during the development of berries in Turpan. For *CHS2*, a higher expression level was found at the later maturation stages. *CHI* had the highest relative expression level at S4 or S5. In addition to the RG−U grapes, *CHI* continued to be upregulated during grape development. Moreover, the expression of *F3H* was slightly upregulated in the middle development stage and presented a downregulation after grape maturation. The relative expression of *F3′H* and *F3′5′H* both presented fluctuating trends. As for *DFR*, the expression level reached its first peak at S5 and its second peak at S1. The expression of *ANS* was upregulated with the development of berries until it was downregulated at S5. Even if there was a slight upregulation of *ANR* in the early stages, the expression of *ANR* was still significantly decreased at the later maturation stages.

The expressions of these key genes are affected both by transcription factors and environmental factors. Light and temperature are the most important environmental factors. The expression of the up-steam genes (including *PAL, 4CL, CHS2, CHI*) of the anthocyanin biosynthesis pathway is kept relatively stable at early developmental stages (S1 to S3), whereas it significantly changes at the mature stages (S4 and S5), in the two varieties of grape berry. The expression of down-steam genes (*DFR* and *ANS*) changed in the early developmental stages of the berries in the different regions. As for *F3H,* as a turning point gene that played an important role for proanthocyanin biosynthesis, the expression was constantly high in the two varieties in the Urumqi region. Previous studies have confirmed that certain light conditions were helpful for the expressions of key genes in the synthesis of anthocyanins in grape berries. Light could positively regulate the expressions of *PAL* and *F3H* (VIT_18s0001g14310), whereas it negatively regulated the expressions of *CHS*, *F3H* (VIT_04s0023g03370), and *F3′5′H* in grapes [16]. The low light conditions caused by bagging could downregulate the expressions of *CHS2*, *CHI*, *F3′H*, *F3′5′H*, *DFR*, and *ANS* in grapes by affecting light-response factors *VvHY5* and *VvCOP1* [17]. However, in our study, the expressions of *PAL*, *4CL*, *DFR*, *F3H*, *F3′5′H*, and *ANS* in grape berries in Turpan with higher temperatures and light intensities were lower. It was speculated that a high temperature inhibited their expressions. A temperature of 35 °C hindered the expressions of *DFR* and *ANS* in grapes when compared with a temperature of 25 °C [13]. High night temperatures inhibited the gene expressions of *CHS*, *F3H*, *DFR*, and *ANS* at the early stage of grape ripening [18]. These studies indicated that most genes related to anthocyanin syntheses were regulated by environmental factors.

### 2.4. Phenolic Compounds, such as the Anthocyanins, of Grape Berries in Urumqi and Turpan

Ten phenolic compounds, including catechin, ferulic acid, epicatechin, and procyanidin B1 and B2, and five anthocyanins, including delphinidin-3-*O*-glucoside, cyanidin-3-*O*-glucoside, petunidin-3-*O*-glucoside, peonidin-3-*O*-glucoside, and malvidin-3-*O*-glucoside, were identified and quantified in grape berries, as shown in Table 1. On the whole, catechin, ferulic acid, epicatechin, and procyanidin B1 and B2 decreased, along with grapes’ development. Interestingly, epicatechin in the two grape varieties showed an increasing trend early on and then decreased in Urumqi berries, with the highest content appearing at S2. For RG grapes, the contents of catechin and ferulic acid in Urumqi berries were higher than those in Turpan berries at S1, S2, S5 and S1, S2, S3, respectively. The procyanidin B1 content of Urumqi grape berries was also higher, although the content of procyanidin B2 among the regions had no obvious regularity. The content of epicatechin was higher in Turpan grape berries at all growing stages, except for S2. For XY grapes, the contents of catechin and ferulic acid in Urumqi berries were significantly higher than those in Turpan. Moreover, Urumqi berries contained more epicatechin, in addition to S1. The measured content of procyanidins was similar with RG grape berries. In general, catechin, ferulic acid, and procyanidin B1 and B2 accumulated more in XY grape berries than in RG grape berries, whereas epicatechin showed a converse pattern.

Catechin, epicatechin, ferulic acids, and procyanidins have been identified in grapes, according to a previous study [1]. Gonzalez et al. also reported that the catechins and procyanidins in plums decreased during fruit ripening [19]. Appropriate light conditions and temperatures are helpful to the synthesis of phenolic compounds in grape berries. The content of flavanols in grape berries grown under shading conditions was lower [7]. Compared with 20 °C, 25 °C was more optimal for the synthesis of flavan-3-ol in ‘Kadainou R-1′ grape berries [20]. In this study, grape berries in Urumqi contained more catechin and ferulic acids. The results showed that the accumulation of phenolic compounds in berries was obviously regulated by temperature under certain light conditions. However, the variation of epicatechin content was diverse due to the different reaction of epicatechin synthesis to temperature and light in different grape varieties. There was no apparent difference in the contents of procyanidin B1 and B2 between Urumqi and Turpan grape berries. A review concluded that the procyanidins in grape skins were regulated by high temperatures during the day, whereas seed composition generally showed very little variation due to abiotic factors [21].

It can be observed that all the anthocyanin compounds in grape berries continued to rise with grape development and reached a maximum at S3 or S4 (Table 1). The contents of delphinidin-3-*O*-glucoside, cyanidin-3-*O*-glucoside, petunidin-3-*O*-glucoside, peonidin-3-*O*-glucoside, and malvidin-3-*O*-glucoside also showed identical trends. During RG grape development, the total anthocyanin contents of Urumqi and Turpan berries ranged from 6.38 ± 0.34 μg/g FW to 38.03 ± 0.68 μg/g FW and 1.19 ± 0.08 μg/g FW to 8.63 ± 0.21 μg/g FW (S4), respectively. From S3 to S5, the contents of cyanidin-3-*O*-glucoside and peonidin-3-*O*-glucoside in Urumqi berries were higher than those in Turpan berries. In Turpan berries, delphinidin-3-*O*-glucoside, petunidin-3-*O*-glucoside, and malvidin-3-*O*-glucoside were not detected during the entire developmental period. During XY grape development, the total anthocyanin contents of Urumqi and Turpan berries significantly increased and reached up to 46.78 ± 1.10 μg/g FW (S3) and 11.68 ± 0.23 μg/g FW (S2), respectively. The changes in anthocyanin accumulation in the two varieties showed the same tendency during berry development in the two regions. Although there were higher detected anthocyanin contents in the RG berries in Urumqi, XY grapes contained more anthocyanin at S1 and S2 when growing in Turpan. The richer anthocyanin content was presented in XY grapes, instead of RG grapes, when growing in Urumqi, which suggested the variety of XY would be more sensitive with climatic changes.

Peonidin-3-*O*-glucoside was the main anthocyanin in both RG and XY grape berries. Apparently, although cultivar-dependent time-course variation was observed for anthocyanin content, in general, the berries of both cultivars grown in Turpan had significant lower anthocyanins than the Urumqi grape berries, which is probably the result of a lower average temperature in Urumqi (Supplementary Figure S1). As reported, the total anthocyanin content of ‘Malbec’ and ‘Bonarda’ grapes under raised temperature conditions for field-crop simulation were significantly lower than their controls [22]. It has been proven that low temperatures greatly enhance anthocyanin accumulation after grape veraison, even if the responses to temperature differed between two cultivars [23]. In addition, a cooling treatment around veraison hastened berries’ anthocyanin accumulation under cool overnight temperatures [11]. However, temperature differences and light intensity are also important environmental factors in affecting the synthesis of plant anthocyanins. Apparently, stronger light intensity and greater temperature differences were observed in Turpan (Supplementary Figure S1). Within a certain temperature range, the higher daytime temperature and appropriate light intensity contribute to stronger photosynthesis, which can provide precursors for anthocyanin synthesis. The anthocyanin accumulation of ‘Cabernet Sauvignon’ grapes increased with increasing light up to 100 mmol·m^−2^·s^−1^ [24]. Recent studies have confirmed that the anthocyanin content of grapes decreased as the light transmittance increased [16]. However, the stronger light in Turpan was not accompanied with a higher anthocyanin content in our study. The accumulation of anthocyanins in plants is regulated by a variety of environmental factors [7]. As early as 2002, it has been demonstrated by research that the accumulation of anthocyanin was more a function of temperature than of light [25].

### 2.5. Antioxidant Activities of Grape Berries in Urumqi and Turpan

**Figure 2:**
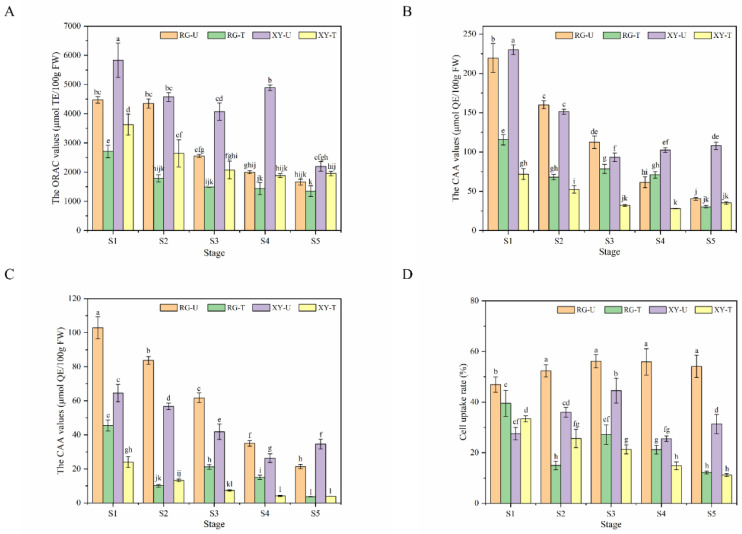
Antioxidant activities of grape berries in ORAC values and CAA values during the five developmental stages in Urumqi and Turpan. (**A**) ORAC values; (**B**) CAA values under no PBS wash protocol; (**C**) CAA values under the PBS wash protocol; (**D**) cell uptake rate. Bars with different letters differ significantly at *p* < 0.05. RG−U, Urumqi ‘Red Globe’ grapes; RG−T, Turpan ‘Red Globe’ grapes; XY−U, Urumqi ‘Xin Yin’ grapes; XY−T, Turpan ‘Xin Yu’ grapes.

We can observe from Figure 2A that the total antioxidant activities of the ORAC values of RG and XY berries decreased continuously during grape maturation in the two regions. However, berries in Urumqi led to significantly higher ORAC values. Variations in cellular antioxidant activity (CAA) values in grape berries are shown in Figure 2B,C. The results were similar with ORAC values. Samples showed maximum cellular antioxidant activities at S1, which then bottomed at S5. Compared to berries in Turpan, the CAA values in Urumqi berries were obviously higher (*p* < 0.05) in each growing stage. In general, the CAA values in the PBS wash protocol were lower than in the no PBS wash protocol. An increasing tendency was presented by the cell uptake ratio in Urumqi berries, whereas a decreasing change was exhibited in Turpan berries (Figure 2D). Moreover, the cell uptake ratios of Urumqi berries were higher than Turpan berries. 

There was a very high correlation between phenolic compounds and antioxidant capacity [26]. Grape berries have been shown to have antioxidant activity by the DPPH assay [27]. In our work, the ORAC assay and CAA assay were applied for measuring the antioxidant activity, and the results showed that the antioxidant activity decreased during maturation. The CAA assay is more biologically relevant than the popular chemistry antioxidant activity assays due to the aspects of cell uptake, metabolism, and the location of antioxidants in cells [28]. The antioxidant activity of grape berries planted in Urumqi was better than in Turpan in both the ORAC assay and the CAA assay. It was speculated that Urumqi grape berries contained more phenolic compounds, particularly anthocyanins. Wang et al. found that the ORAC and CAA values of blueberries with lower phenolic content were worse [29]. The relationship between anthocyanins and antioxidant ability has been demonstrated previously [27].

### 2.6. Cytotoxicity and the Anti-Proliferative Activities of Grape Berries in Urumqi and Turpan

The CC_50_ value of grape berry extracts was high, with values up to 80 mg/mL (the results are not shown). This result illustrated that the grape berry extracts showed no cytotoxicity at the concentrations tested for anti-proliferative activity. The anti-proliferative activities of the grape berries expressed by the EC_50_ value are shown in Supplementary Table S2. The anti-proliferative activity of grape berries declined regardless of the variety and regions during grape ripening. Urumqi berries had higher anti-proliferative activity than Turpan berries. 

A number of previous reports indicated that a high consumption of grape berries’ phenolic compounds could be associated with a reduced risk of cancers [30], due to the mechanisms by which grape berry antioxidants can exert potential anti-cancer effects, including antioxidant, anti-inflammatory, and anti-proliferative activities. In our study, grape berries were reported to have an anti-proliferative effect on HepG2 cells. Urumqi berries contained higher phenolics and anthocyanins had better anti-proliferative activity. Different blueberry extracts could inhibit HepG2 proliferation and maintain a positive correlation with changes in anthocyanin content [29]. Anthocyanins in strawberries could play a role in the prevention and treatment of breast cancer, which could eventually trigger the apoptosis of breast cancer cells by inhibiting the proliferation and metastasis of cancer cells [31].

### 2.7. Correlation Analysis

**Figure 3:**
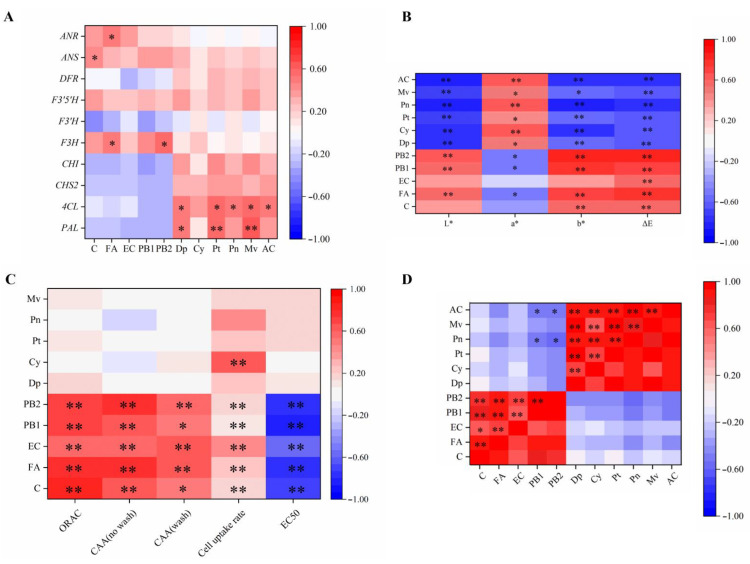
Pearson’s correlation analysis in grape berries during the five developmental stages in Urumqi and Turpan. (**A**) Gene expression and composition; (**B**) composition and color difference values; (**C**) composition and antioxidant quality; (**D**) composition. C, FA, EC, PB1, PB2, Dp, Cy, Pt, Pn, Mv, and AC stand for catechin, ferulic acid, epicatechin, procyanidin B1, procyanidin B2, delphinidin-3-*O*-glucoside, cyanidin-3-*O*-glucoside, petunidin-3-*O*-glucoside, peonidin-3-*O*-glucoside, mal-vidin-3-*O*-glucoside, and total anthocyanins, respectively. *, Significant at 5% probability levels; **, significant at 1% probability levels.

A Pearson’s correlation analysis of the components in grape berries and the key genes of their synthetic pathways are shown in Figure 3A. The transcript levels of *F3H*, *F3′5′H*, *ANS*, and *ANR* exhibited positive correlations with phenolics, whereas most relative expressions of genes showed obvious positive correlations with anthocyanins. *PAL* and *4CL* were speculated to be the main genes regulating grape berries’ anthocyanin synthesis, since they were significantly related to most anthocyanin components, which would be more sensitive with environmental stresses, particularly growth temperature during berry ripening. The Pearson’s correlation analysis of grape berry components and color difference are shown in Figure 3B. Moreover, significant positive correlations were observed between anthocyanins and a* values, whereas anthocyanin contents were significantly negatively associated with L* and b*. It was also verified that anthocyanins were the important coloring substances for the red color of grape berries. The Pearson’s correlation analysis of the phenolic components and activities of grape berries is shown in Figure 3C. The CAA values with PBS wash protocol, the CAA values with no PBS wash protocol, and ORAC values exhibited significant positive correlations with phenolic compounds, although there was no obvious correlation between their values and anthocyanins. The cell uptake rate and antiproliferative activity were positively correlated with phenolics, including anthocyanins. The Pearson’s correlation analysis between grape berry components is shown in Figure 3D. There were strong correlations among phenolics. Moreover, anthocyanins were correlated with each other. Viewing the relationships of total anthocyanins (AC), procyanidin B1 and B2 were negatively related with AC, whereas all kinds of anthocyanins were simultaneously highly correlated with AC.

### 2.8. Principal Component Analysis (PCA) and Cluster Analysis in Grape Berries

**Figure 4:**
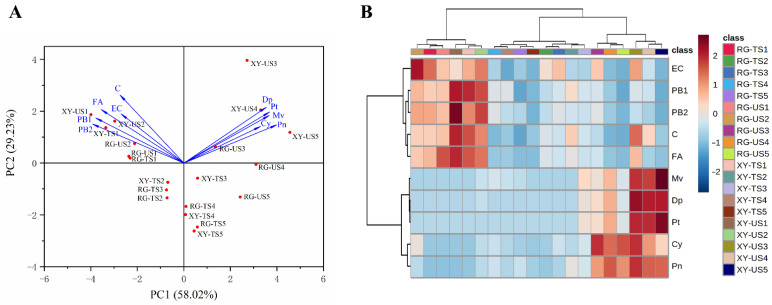
Principal component analysis (**A**) and cluster analysis (**B**) in grape berries during the five developmental stages in Urumqi and Turpan. RG−U, Urumqi ‘Red Globe’ grapes; RG−T, Turpan ‘Red Globe’ grapes; XY−U, Urumqi ‘Xin Yin’ grapes; XY−T, Turpan ‘Xin Yu’ grapes. RG−US1, RG−US2, RG−US3, RG−US4, RG−US5, XY−US1, XY−US2, XY−US3, XY−US4, RG−US5, RG−TS1, RG−TS2, RG−TS3, RG−TS4, RG−TS5, XY−TS1, XY−TS2, XY−TS3, XY−TS4, and RG−TS5 stand for grape berry samples. C, FA, EC, PB1, PB2, Dp, Cy, Pt, Pn, and Mv stand for catechin, ferulic acid, epicatechin, procyanidin B1, procyanidin B2, delphinidin-3-*O*-glucoside, cyanidin-3-*O*-glucoside, petunidin-3-*O*-glucoside, peonidin-3-*O*-glucoside, and malvidin-3-*O*-glucoside, respectively.

A PCA was conducted to further compare the effects of different growth conditions on grape berry components (Figure 4A). In grape berries, the contribution values of PC1 and PC2 were 58.02% and 29.23%, respectively. Grape berries in later development stages were classified into first and fourth quadrants, and berries in S1 and S2 were located in the second and third quadrants. As previously shown, anthocyanins were the more prominent components in Urumqi grape berries, whereas phenolics were more prominent in S1 grape berries. The results further confirmed that Urumqi grape berries contained more anthocyanins.

The 20 grape berry samples were divided into three groups by component clustering (Figure 4B). The first group included RG−TS1, RG−US1, RG−US2, XY−TS1, XY−US1, and XY−US2, which were mainly early grape berries with higher phenolic compound contents in Turpan and Urumqi; the other samples were clustered into the second group and were subdivided into two subgroups according to growing area. Predominantly, samples grown in Urumqi contained more anthocyanins. The results of the cluster analysis showed that grape berry compositions from the two regions were basically similar at early grape development stages. At later stages of grapes development, the content of anthocyanins in Urumqi berries was higher than Turpan berries. The results showed more abundant phenolic compounds in early grape berries; moreover, they indicated that the environment had a significant impact on the composition of grape berries, and that the accumulation of grape anthocyanins in areas with lower temperatures and light intensity were significantly higher.

## 3. Materials and Methods

### 3.1. Materials

Both ‘Red Globe’(RG) and ‘Xin Yu’(XY) grape berries in the five developmental stages (S1, S2, S3, S4, and S5) were selected in the experiment bases of the Xinjiang Institute of Agricultural Sciences in Turpan and Urumqi, Xinjiang, China. Urumqi and Turpan grape berries were collected for the first time in 47 and 45 days after flowering, respectively. Other sampling times are shown in Supplementary Table S3. Photographs of picked grape berries at each developmental stage from the different regions are shown in Supplementary Figure S1. The grape berries were divided into four groups: RG-U, or Urumqi ‘Red Globe’ grapes; RG-T, or Turpan ‘Red Globe’ grapes; XY-U, or Urumqi ‘Xin Yin’ grapes; XY-T, or Turpan ‘Xin Yu’ grapes. Each group included 15 stocks with the same shape and each stock retained 15 clusters of berries. Five to ten strings were sampled at each stage; 600 g berries with the same size, uniform color, no diseases, and no pests were weighted three times for biological parallelism. The samples were collected and quickly frozen in liquid nitrogen. All the grape berries were stored at −80 °C until analysis.

### 3.2. Reagent and Chemicals

Catechin, ferulic acid, epicatechin, quercetin, 2,2′-azobis-amidinopropane (ABAP), Trolox, and Folin–Ciocalteu reagent were purchased from Sigma (St. Louis, MO, USA). Procyanidin B1, procyanidin B2, delphinidin-3-*O*-glucoside, cyanidin-3-*O*-glucoside, petunidin-3-*O*-glucoside, peonidin-3-*O*-glucoside, and malvidin-3-*O*-glucoside were purchased from Weikeqi Biological Technology Co., Ltd. (Chengdu, Sichuan Province, China). HPLC-grade agents were purchased from ANPEL Scientific Instrument Co., Ltd. (Shanghai, China). Williams’ medium E, PBS, and trypsin-EDTA solution were purchased from Gibco (Life Technologies, Grand Island, NY, USA). The human liver cancer cell line HepG2 (ATCC HB-8065) was purchased from ATCC Company (Manassas, VA, USA). Other chemicals and reagents used in this work were analytical grade.

### 3.3. Determination of Color Difference Value

The color of grape berries was determined by a colorimeter (Chroma Meter II Reflectance CR-300 triple, Konica Minolta Holdings, Inc., Osaka, Japan). The color difference values of three parameters were measured to characterize the color of grape berries: (1) the brightness value L*, with a larger L* meaning a higher brightness; otherwise, it is darker; (2) the redness–greenness value a*, with −a* meaning green and +a* meaning red; and (3) the yellowness–blueness value b*, with −b* indicating blue and +b* indicating yellow. The total color difference values were calculated by the following formula:

$$\Delta E=\sqrt{{\left(L-L_{0}\right)}^{2}+{\left(a-a_{0}\right)}^{2}+{\left(b-b_{0}\right)}^{2}}.$$

### 3.4. Total RNA Extraction, Reverse Transcription and RT-qPCR Analysis

The total RNA of grape berries was extracted with the RNAprep Pure Plant Kit (DP441) (Tiangen, Beijing, China) and reverse transcribed to cDNA using the FastKing gDNA Dispelling RT SuperMix. RT-qPCR was conducted with SuperReal PreMix Plus (SYBR Green), referring to the manufacturer’s instructions (Tiangen, Beijing, China), and was accomplished using the LightCycler^®^ 480 Real-Time PCR System (F.Hoffmann-La Roche Ltd, Switzerland). The nucleotide sequences of the primers used in the RT-qPCR are presented in Supplementary Table S4. Ubiquitin was regarded as the reference gene. The 2^−ΔΔCt^ method based on three technical replicates was used to analyze the transcript levels.

### 3.5. Phenolic Substance Extracts of Grape Berries

Phenolic substances of grapes were extracted by the following method, as reported previously [32]. Briefly, triplicate 20 g were extracted with 200 mL of methanol/glacial acetic acid (5:1, *v*/*v*). The extracts were kept at 4 °C overnight after homogenization at a rate of 12,000 rpm for 2 min. All of the extracts were evaporated at 45 °C and redissolved with methanol and maintained at −20 °C for analysis.

### 3.6. Determination of Phenolic Substances, Such as Anthocyanins

Phenolic substances, such as anthocyanins, were analyzed by HPLC with a photodiode array detector (Waters, LC 2998, Milford, MA, USA) with a C18 column (Waters, 250 * 4.6 mm, 5 μm) maintained at 35 °C, as per a previous experimental analysis [32]. Briefly, the analysis of phenolic compounds was performed at a wavelength of 280 nm with 1.0 mL/min of the mobile phases (A, 0.1% trifluoroacetic acid in water; B, acetonitrile) in gradient elution as follows: from 0 to 5 min, 90% A; from 5 to 20 min, 90% to 75% A; from 20 to 25 min, 75% to 65% A; from 25 to 31 min, 65% to 42% A; from 31 to 34 min, 42 to 40% A; from 34 to 40 min, 40% to 10% A; from 40 to 50 min, 10% to 90% A; from 50 to 53 min, 90% A. The values were presented as “mg/100 g FW”. Additionally, the analysis of anthocyanins was performed at 520 nm with 1.0 mL/min of the mobile phases as follows: from 0 to 5 min, 90% A; from 5 to 25 min, 90% to 75% A; from 25 to 27 min, 75% to 65% A; from 27 to 30 min, 65% to 10% A; from 30 to 32 min, 10% to 90% A; from 32 to 37 min, 90% A. The values were presented as “μg/g FW”. The data were reported as the mean ± SD (*n* = 3) for triplicate analysis.

### 3.7. Determination of Antioxidant Activities

Antioxidant activity in vitro was determined by an oxygen radical absorbance capacity (ORAC) assay and presented as µmol Trolox equivalent per 100 g of fresh weight (µmol TE/100 g FW) [33]. Trolox was used as the standard. The antioxidant activity of grape berries was also determined by a cellular antioxidant activity (CAA) assay and presented as μmol quercetin equivalent per 100 g of fresh weight (μmol QE/100 g FW) [28]. Quercetin was used as the standard. The data were reported as the mean ± SD (*n* = 3) for triplicate analysis.

### 3.8. Determination of Cytotoxicity Assay and Anti-Proliferation Activity

The cytotoxicity activity and anti-proliferative activity of grape berries were detected with the methods as described previously [34,35]. The methods of culturing cells were similar to the methods used for the CAA assay. The data were reported as the mean ± SD (*n* = 3) for triplicate analysis. The cell anti-proliferation activity and the cytotoxicity analysis of the samples were expressed in EC_50_ mg/mL and CC_50_ mg/mL, respectively.

### 3.9. Statistical Analysis

Data were expressed as mean ± SD for triplicate analysis. Results were evaluated applying ANOVA statistical analysis with Duncan’s multiple range test by using IBM SPSS 25.0 (IBM, Armonk, NY, USA). Correlation tests and cluster analysis were performed using the methodology of the Pearson’s correlation coefficient. The mean-centered and scaled data were used for principal component analysis (PCA) to clarify the relationship between grape berry components of different cultivars and developmental stages.

## 4. Conclusions

In summary, we evaluated anthocyanin accumulation and the antioxidant activity of two kinds of grape berries growing in various climate conditions during five natural stages. Climate had significant effects on the anthocyanin metabolism and antioxidant quality of grape berries. The climate condition in Urumqi, with lower light intensity, temperatures, and humidity, resulted in a higher redness of grapes during development and was supposed to be more beneficial for the accumulation of phenolic substances, especially anthocyanins. The antioxidant activity of grape berries grown in Urumqi was greater than Turpan grape berries. These results contribute to the general understanding of the effect of climate conditions on anthocyanin accumulation and the quality formation of grapes and, thus, could contribute to grape cultivation, collection, and processing.

## Figures and Tables

**Table 1:** Changes in phenolic and anthocyanin profiles in grape berries during the five developmental stages in Urumqi and Turpan (S1, S2, S3, S4, and S5).

Stage	Groups	C mg/100 g FW	FA mg/100 g FW	EC mg/100 g FW	PB1 mg/100 g FW	PB2 mg/100 g FW	Dp μg/g FW	Cy μg/g FW	Pt μg/g FW	Pn μg/g FW	Mv μg/g FW
S1	RG−U	26.50 ± 1.97^e^	13.28 ± 0.20^b^	16.36 ± 0.62^e^	6.30 ± 0.19^e^	3.50 ± 0.02^e^	ND	ND	ND	ND	ND
	RG−T	24.68 ± 0.79^f^	9.20 ± 0.23^d^	21.87 ± 0.35^b^	6.18 ± 0.15^e^	3.91 ± 0.01^d^	ND	ND	ND	ND	ND
	XY−U	45.32 ± 1.05^a^	14.92 ± 0.31^a^	14.11 ± 0.21^f^	9.91 ± 0.30^a^	6.69 ± 0.07^a^	ND	ND	ND	ND	ND
	XY−T	40.24 ± 0.54^b^	13.20 ± 0.11^b^	17.94 ± 2.02^d^	9.58 ± 0.01^b^	4.41 ± 0.10^c^	ND	0.02 ± 0.00^l^	ND	0.47 ± 0.01^n^	ND
S2	RG−U	24.37 ± 0.31^fg^	8.37 ± 0.06^f^	27.23 ± 0.43^a^	6.69 ± 0.20^d^	3.95 ± 0.03^d^	ND	1.27 ± 0.07^f^	ND	5.11 ± 0.27^i^	ND
	RG−T	16.69 ± 0.27^h^	5.29 ± 0.01^h^	13.81 ± 0.12^f^	4.00 ± 0.01^j^	1.78 ± 0.00^jk^	ND	0.12 ± 0.01^k^	ND	1.07 ± 0.07^m^	ND
	XY−U	34.10 ± 0.47^d^	12.03 ± 0.07^c^	21.06 ± 0.23^b^	8.92 ± 0.23^c^	5.37 ± 0.03^b^	0.06 ± 0.00^g^	0.54 ± 0.02^i^	ND	5.16 ± 0.20^i^	0.54 ± 0.02^i^
	XY−T	25.17 ± 0.19^f^	6.05 ± 0.05^g^	9.03 ± 0.29^h^	5.62 ± 0.01^f^	1.96 ± 0.01^h^	ND	0.42 ± 0.01^j^	ND	11.25 ± 0.22^f^	ND
S3	RG−U	12.50 ± 0.85^j^	4.79 ± 0.12^i^	16.00 ± 0.93^e^	4.55 ± 0.05^h^	2.36 ± 0.00^f^	0.24 ± 0.02^d^	3.99 ± 0.19^a^	0.52 ± 0.03^d^	23.93 ± 1.08^e^	1.42 ± 0.06^f^
	RG−T	14.54 ± 0.01^i^	4.72 ± 0.05^i^	16.65 ± 0.09^e^	4.52 ± 0.05^h^	2.27 ± 0.01^g^	ND	0.71 ± 0.04^h^	ND	3.35 ± 0.17^k^	ND
	XY−U	37.24 ± 0.51^c^	8.97 ± 0.19^e^	18.99 ± 0.54^c^	4.72 ± 0.03^g^	1.80 ± 0.02^j^	0.86 ± 0.02^a^	3.99 ± 0.10^a^	1.57 ± 0.03^b^	35.63 ± 0.86^a^	4.74 ± 0.09^b^
	XY−T	15.20 ± 0.46^i^	4.63 ± 0.15^i^	8.59 ± 0.67^hi^	4.44 ± 0.01^hi^	1.89 ± 0.01^i^	0.20 ± 0.01^e^	0.82 ± 0.03^d^	0.47 ± 0.01^e^	8.13 ± 0.21^g^	1.81 ± 0.04^e^
S4	RG−U	5.09 ± 0.15^lm^	2.17 ± 0.04^l^	6.33 ± 0.09^j^	3.28 ± 0.02^k^	1.19 ± 0.03^n^	0.41 ± 0.00^c^	3.25 ± 0.06^c^	0.88 ± 0.02^c^	31.03 ± 0.56^b^	2.46 ± 0.03^d^
	RG−T	5.90 ± 0.03^l^	2.49 ± 0.03^k^	8.40 ± 0.05^hi^	4.75 ± 0.05^g^	2.30 ± 0.14^g^	ND	0.65 ± 0.01^h^	ND	7.18 ± 0.18^h^	0.80 ± 0.02^h^
	XY−U	23.71 ± 0.27^c^	3.11 ± 0.03^j^	10.40 ± 0.05^g^	4.32 ± 0.11^i^	1.38 ± 0.03^m^	0.79 ± 0.02^b^	2.66 ± 0.05^d^	1.55 ± 0.03^b^	30.23 ± 0.55^c^	4.49 ± 0.03^c^
	XY−T	8.28 ± 0.59^k^	2.65 ± 0.03^k^	4.83 ± 0.05^k^	4.84 ± 0.02^g^	1.73 ± 0.01^k^	ND	0.38 ± 0.01^j^	ND	3.90 ± 0.05^j^	0.50 ± 0.00^i^
S5	RG−U	4.32 ± 0.33^m^	1.74 ± 0.10^m^	5.39 ± 0.20^jk^	2.36 ± 0.16^mn^	0.76 ± 0.04^o^	0.13 ± 0.00^f^	3.54 ± 0.10^b^	0.32 ± 0.00^f^	25.26 ± 0.61^d^	0.86 ± 0.01^g^
	RG−T	2.81 ± 0.23^n^	1.80 ± 0.08^m^	7.59 ± 0.43^i^	2.25 ± 0.01^n^	1.54 ± 0.00^l^	ND	0.16 ± 0.00^k^	ND	6.84 ± 0.24^h^	0.34 ± 0.01^j^
	XY−U	8.74 ± 0.12^k^	2.11 ± 0.05^l^	5.72 ± 0.10^jk^	2.43 ± 0.01^m^	0.67 ± 0.04^p^	0.79 ± 0.03^b^	1.74 ± 0.02^e^	1.87 ± 0.04^a^	29.74 ± 0.66^c^	6.24 ± 0.10^a^
	XY−T	8.11 ± 0.36^k^	1.85 ± 0.04^m^	5.59 ± 0.22^jk^	2.73 ± 0.00^l^	0.69 ± 0.01^p^	ND	0.03 ± 0.00^l^	ND	2.31 ± 0.05^l^	ND

Means with different letters differ significantly in each column at *p* < 0.05. “ND” means that values are not quantifiable. RG−U, ‘Red Globe’ grapes in Urumqi; RG−T, ‘Red Globe’ grapes in Turpan; XY−U, ‘Xin Yu’ grapes in Urumqi; XY−U, ‘Xin Yu’ grapes in Turpan. C, catechin; FA, ferulic acid; EC, epicatechin; PB1, procyanidin B1; PB2, procyanidin B2; Dp, delphinidin-3-*O*-glucoside; Cy, cyanidin-3-*O*-glucoside; Pt, petunidin-3-*O*-glucoside; Pn, peonidin-3-*O*-glucoside; Mv, malvidin-3-*O*-glucoside.

## Data Availability

The data presented in this study are available in this article and supplemental files.

## Supplementary Materials

The following supporting information can be downloaded online. Figure S1. Climatic conditions in Urumqi and Turpan from 2 July 2018 to 14 September 2018; Figure S2. Photographs of grape berries during the five developmental stages in Urumqi and Turpan; Table S1. The color difference values of grape berries during the five developmental stages in Urumqi and Turpan (L*, a*, b* and ΔE); Table S2. Anti-proliferative activity of grape berries during the five developmental stages in Urumqi and Turpan (S1, S2, S3, S4 and S5); Table S3. Sampling time of grape berries during the five developmental stages in Urumqi and Turpan; Table S4. The reference gene and primers used.
