# Supplementary material for: Dynamic Changes in Anthocyanin Accumulation and Cellular Antioxidant Activities in Two Varieties of Grape Berries during Fruit Maturation under Different Climates
Source: Molecules. 2022 Jan 7;27(2):384. doi: 10.3390/molecules27020384 (PMC8782009; doi:10.3390/molecules27020384)
Supplement: Supplementary file 1 [file molecules-27-00384-s001.zip › molecules-1487714-supplementary.pdf]

Supplementary Materials

Dynamic Changes in Anthocyanin Accumulation and Cellular Antioxidant Activities in Two Varieties of Grape Berries during Fruit Maturation under Different Climates

Liwei Qin [1,†], Hui Xie [2,†], Nan Xiang [1], Min Wang [2], Shouan Han [2], Mingqi Pan [2], Xinbo Guo [1,*], Wen Zhang [2,*]

[1] Engineering Research Center of Starch and Vegetable Protein Processing Ministry of Education, Guangdong Province Key Laboratory for Green Processing of Natural Products and Product Safety, School of Food Science and Engineering, South China University of Technology, Guangzhou 510640, China; qlw13710614008@163.com (L.Q.); nanxiang0908@163.com (N.X.)

[2] Research Institute of Horticulture, Key Laboratory of Genome Research and Genetic Improvement of Xinjiang Characteristic Fruits and Vegetables, Xinjiang Academy of Agricultural Sciences, Urumqi 830091, China; xhxjnky@163.com (H.X.); wangmin_807032699@163.com (M.W.); hanshouan@163.com (S.H.); panmq3399@sohu.com (M.P.)

† These authors contributed equally.

* Correspondence: guoxinbo@scut.edu.cn (X.G.); zwxilin@126.com (W.Z.); Tel./Fax: +8620-87113848 (X.G.); +86991-4503409 (W.Z.)

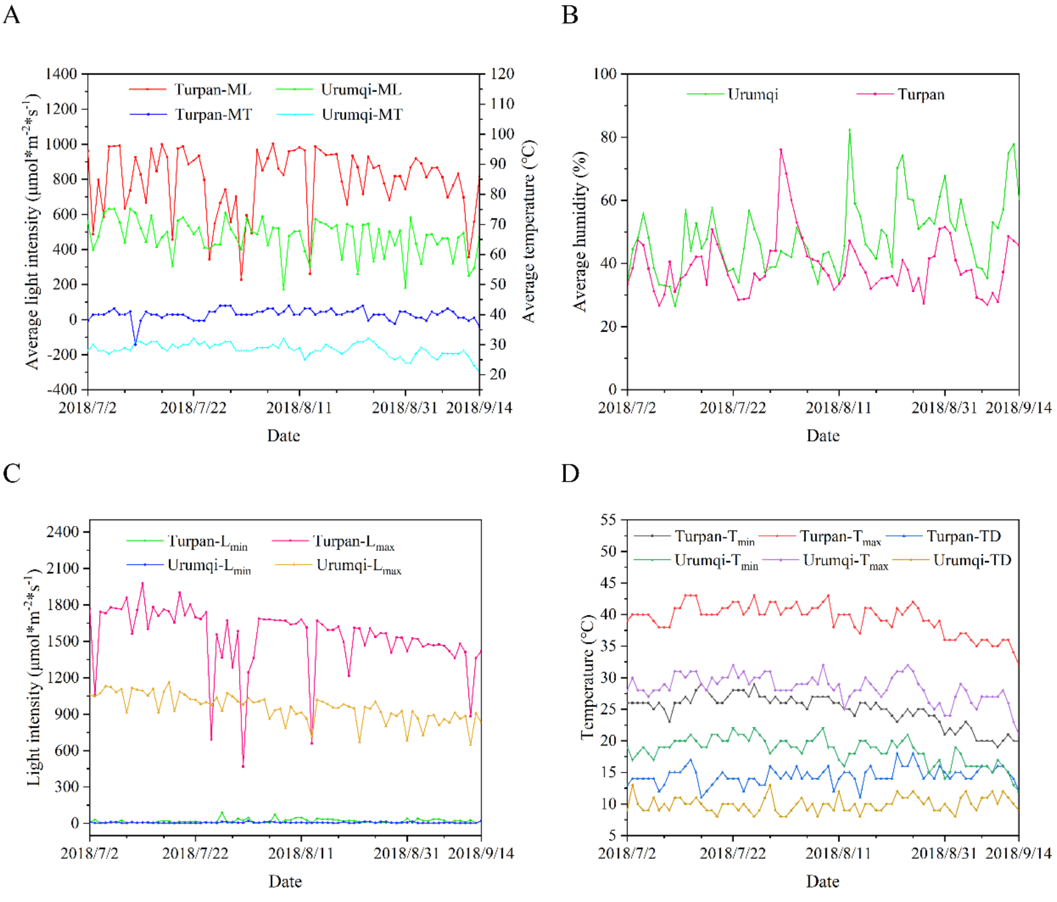

Figure S1. Climatic conditions in Urumqi and Turpan from 2 July, 2018 to 14 September, 2018. (A) Average daily temperature and light intensity. (B) Average daily humidity. (C) Maximum and minimum light intensity. (D) Temperature difference, maximum and minimum temperature. ML: average light intensity; MT: average temperature; L_{min}: minimum light intensity; L_{max}: maximum light intensity; T_{min}: minimum temperature; T_{max}: maximum temperature, TD: temperature difference.

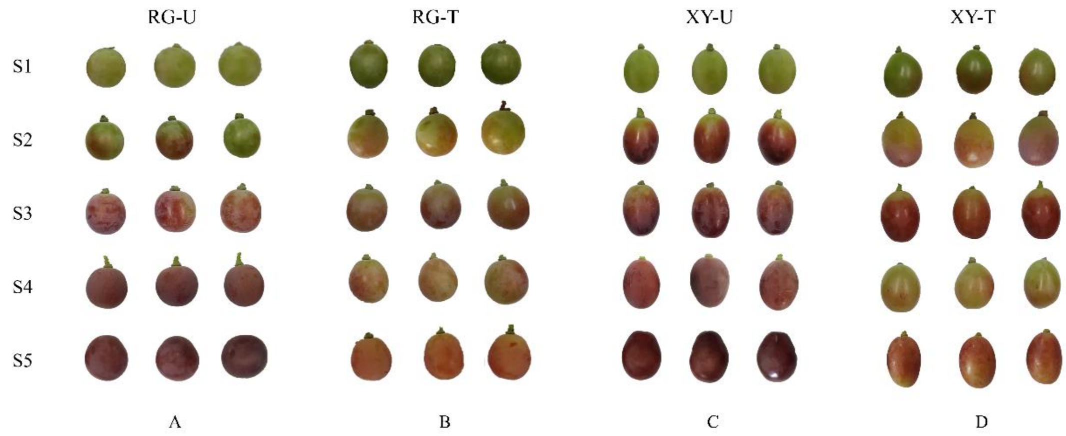

Figure S2. Photographs of grape berries during the five developmental stages in Urumqi and Turpan. (A) 'Red Globe' grapes in Urumqi; (B) 'Red Globe' grapes in Turpan; (C) 'Xin Yu' grapes in Urumqi; (D) 'Xin Yu' grapes in Turpan.

Table S1. The color difference values of grape berries during the five developmental stages in Urumqi and Turpan (L*, a*, b* and ΔE).

Stage	Groups	L*	a*	b*	ΔE
S1	RG–U	22.95	2.31	12.50	26.69
	RG–T	20.90	−3.63	14.09	25.69
	XY–U	25.26	−4.04	20.62	32.78
	XY–T	18.05	−0.37	10.95	21.14
S2	RG–U	17.55	5.87	8.33	22.54
	RG–T	17.13	1.59	10.97	20.59
	XY–U	10.63	8.55	14.52	20.51
	XY–T	14.99	5.14	9.01	18.04
S3	RG–U	9.03	17.25	0.70	20.16
	RG–T	15.85	6.55	8.56	19.56
	XY–U	2.55	14.12	0.63	14.53
	XY–T	15.05	4.58	8.97	18.54
S4	RG–U	6.75	5.25	-0.93	8.89
	RG–T	14.81	8.06	7.36	18.82
	XY–U	5.04	9.80	2.21	11.62
	XY–T	15.61	3.89	8.90	19.28
S5	RG–U	6.80	6.74	1.45	8.16
	RG–T	12.52	11.62	4.46	18.81
	XY–U	7.05	8.74	4.43	8.58
	XY–T	9.86	7.86	6.04	14.62

RG–U, 'Red Globe' grapes in Urumqi; RG–T, 'Red Globe' grapes in Turpan; XY–U, 'Xin Yu' grapes in Urumqi; XY–U, 'Xin Yu' grapes in Turpan. +L* = lightness, –L* = brightness, +a* = redness, –a* = greenness, +b* = yellowness, – b* = blueness, ΔE = total aberrations.

Table S2. Anti-proliferative activity of grape berries during the five developmental stages in Urumqi and Turpan (S1, S2, S3, S4 and S5).

	Groups	S1	S2	S3	S4	S5
Anti-proliferative activity in EC50 value (mg/mL)	RG–U	20.84 ± 0.08^{g}	21.59 ± 0.20^{g}	29.12 ± 0.81^{e}	26.86 ± 1.19^{f}	27.54 ± 1.36^{ef}
	RG–T	27.49 ± 0.28^{ef}	37.03 ± 1.12^{c}	27.96 ± 0.82^{ef}	33.12 ± 1.37^{d}	47.47 ± 2.51^{a}
	XY–U	12.63 ± 0.58^{i}	18.07 ± 0.98^{h}	28.74 ± 2.08^{ef}	27.19 ± 1.42^{ef}	38.68 ± 1.38^{bc}
	XY–T	21.01 ± 0.49^{c}	32.07 ± 2.50^{d}	32.13 ± 0.21^{d}	32.11 ± 0.33^{d}	39.17 ± 0.27^{b}

Means with different letters differ significantly index $p < 0.05$. RG–U, 'Red Globe' grapes in Urumqi; RG–T, 'Red Globe' grapes in Turpan; XY–U, 'Xin Yu' grapes in Urumqi; XY–U, 'Xin Yu' grapes in Turpan.

Table S3. Sampling time of grape berries during the five developmental stages in Urumqi and Turpan.

Regions	Date	Stage
Urumqi	2018/7/22	S1
	2018/8/4	S2
	2018/8/19	S3
	2018/9/3	S4
	2018/9/14	S5
Turpan	2018/7/5	S1
	2018/7/20	S2
	2018/8/4	S3
	2018/8/18	S4
	2018/9/3	S5

Table S4. The reference gene and primers used.

Gene name	Fene(bank) ID	Prime sequence (from 5′ to 3′)
PAL	JN858957.1	F: ACAACAATGGACTGCCATCA<br>R: GGGAGGAGATTAAGCCCAAG
4CL	JN858959.1	F: GTCCCGCAAAAAGATGATGT<br>R: TGACTTTGGAATGGCATGAA
CHS2	100232843	F: ACCCACCTTGTTTTCTGCAC<br>R: CAGAGCAGACGACCAAAACA
CHI	100233078	F: GTCTCAAGTGCCGTCAGTCA<br>R: AACCGAATCCGCTAACTCCT
F3H	100233079	F: ATGTCTGGTGGCAAGAAAGG<br>R: CTCAGACAACACCTCCAGCA
F3′H	100232999	F: TGCCCCAACTCACCTATCTC<br>R: TATGGCCCACACGTTCACTA
F3′5′H	100261319	F: AAGCGTGCTCACGAAGAAAT<br>R: TTCCCAGACATCAGGGTCTC
DFR	100233141	F: GCGAGAGCGTAGAAATGTCC<br>R: TGGGCCATTCCGTTTTATTA
ANS	100233142	F: CAAGCTTGCCAACAATGCTA<br>R: CGTTGGAGGAAGAACCTCAG
ANR	100232981	F: CTTGATGGGACAGGTCTGGT<br>R: GTAGCTCTTCCATCCCACCA
UBI	100248856	F: AATCCAGCGATACCATCGAC<br>R: CGAACCAGATGCAGAGTTGA
